# Complementary, alternative and integrative medicine for autism: an umbrella review and online platform

**DOI:** 10.1038/s41562-025-02256-9

**Published:** 2025-08-28

**Authors:** Corentin J. Gosling, Laure Boisseleau, Marco Solmi, Micheal Sandbank, Lucie Jurek, Mikail Nourredine, Gabriella Porcu, Elisa Murgia, Joaquim Radua, Paolo Fusar-Poli, Klara Kovarski, Serge Caparos, Ariane Cartigny, Samuele Cortese, Richard Delorme

**Affiliations:** 1 DysCo Laboratory, Paris Nanterre University, Nanterre, France; 2 Department of Child and Adolescent Psychiatry, Robert Debré Hospital, APHP, Paris, France; 3 Developmental EPI (Evidence Aynthesis, Prediction, Implementation) Lab, Centre for Innovation in Mental Health, School of Psychology, Faculty of Environmental and Life Sciences, University of Southampton, Southampton, UK; 4 SCIENCES lab, Department of Psychiatry, University of Ottawa, Ottawa, Ontario Canada; 5 Department of Mental Health, The Ottawa Hospital, Ottawa, Ontario Canada; 6 Ottawa Hospital Research Institute (OHRI), Ottawa, Ontario Canada; 7 Department of Child and Adolescent Psychiatry, Charité Universitätsmedizin, Berlin, Germany; 8 Department of Health Sciences, School of Medicine, University of North Carolina at Chapel Hill, Chapel Hill, NC USA; 9 Pôle de psychiatrie de l’enfant et de l’adolescent, CH Le Vinatier, Bron, France; 10 Service de biostatistique-bioinformatique, Hospices Civils de Lyon, Lyon, France; 11 Imaging of Mood- and Anxiety-Related Disorders Group, Institut d’Investigacions Biomèdiques August Pi i Sunyer, CIBERSAM, University of Barcelona, Barcelona, Spain; 12 Early Psychosis: Interventions and Clinical-detection (EPIC) Lab, Department of Psychosis Studies, Institute of Psychiatry, Psychology and Neuroscience, King’s College London, London, UK; 13 Department of Brain and Behavioral Sciences, University of Pavia, Pavia, Italy; 14 Outreach and Support in South-London (OASIS) Service, South London and Maudsley NHS Foundation Trust, London, UK; 15 Department of Psychiatry and Psychotherapy, University Hospital, Ludwig Maximilian University, Munich, Germany; 16 Sorbonne Université, Faculté des Lettres, INSPE, Paris, France; 17 LaPsyDÉ, CNRS, Université Paris Cité, Paris, France; 18 DysCo Lab, Université Paris 8, Saint-Denis, France; 19 Institut Universitaire de France, Paris, France; 20 Laboratoire de Psychopathologie et Processus de Santé, Université Paris Cité, Boulogne-Billancourt, France; 21 Clinical and Experimental Sciences (CNS and Psychiatry), Faculty of Medicine, University of Southampton, Southampton, UK; 22 New York University Child Study Center, Hassenfeld Children’s Hospital at NYU Langone, New York City, NY USA; 23 DiMePRe-J – Department of Precision and Regenerative Medicine, Jonic Area, University of Bari ‘Aldo Moro’, Bari, Italy; 24 Human Genetics and Cognitive Functions, Institut Pasteur, Paris, France; 25 Université Paris Cité, Paris, France

**Keywords:** Therapeutics, Careers, Psychology

## Abstract

The use of complementary, alternative and integrative medicine (CAIM) is highly prevalent among autistic individuals, with up to 90% reporting having used CAIM at least once in their lifetime. However, the evidence base for the effects of CAIM for autism remains uncertain. Here, to fill this gap, we conducted an umbrella review of meta-analyses exploring the effects of CAIM in autism across the lifespan and developed a web platform to disseminate the generated results. Five databases were searched (up to 31 December 2023) for systematic reviews with meta-analyses exploring the effects of CAIM in autism. Independent pairs of investigators identified eligible papers and extracted relevant data. Included meta-analyses were reestimated using a consistent statistical approach, and their methodological quality was assessed with AMSTAR-2. The certainty of evidence generated by each meta-analysis was appraised using an algorithmic version of the GRADE framework. This process led to the identification of 53 meta-analytic reports, enabling us to conduct 248 meta-analyses exploring the effects of 19 CAIMs in autism. We found no high-quality evidence to support the efficacy of any CAIM for core or associated symptoms of autism. Although several CAIMs showed promising results, they were supported by very low-quality evidence. The safety of CAIMs has rarely been evaluated, making it a crucial area for future research. To support evidence-based consideration of CAIM interventions for autism, we developed an interactive platform that facilitates access to and interpretation of the present results (https://ebiact-database.com).

## Abstract (web summary)

The use of complementary, alternative and integrative medicine (CAIM) is highly prevalent among autistic individuals. This umbrella review finds that there is no high-quality evidence to support the efficacy of any CAIM for core or associated symptoms of autism. Although several CAIMs showed promising results, they were supported by very low-quality evidence.

## Main

Autism spectrum disorder, hereafter referred to as autism, is a neurodevelopmental condition characterized by impairments in communication and social interaction, as well as restricted, stereotyped and repetitive behaviours and/or interests, frequently associated with sensory differences, all of which interfere with quality of life^1^. Note that, recognizing the preference of most individuals with lived experience on the autism spectrum, this Article adopts identity-first language (for example, ‘autistic individuals’), which places references to the autism spectrum at the forefront of statements. We also acknowledge that some individuals diagnosed with autism spectrum disorder prefer person-first language, which prioritizes mentioning the person before the condition.

Interestingly, although not specifically designed for autism, many autistic individuals utilize complementary, alternative and integrative medicine (CAIM)^2^. Several factors may contribute to this choice. For example, personal values, the perceived lack of efficacy of conventional interventions, and challenges in accessing or implementing traditional interventions may all encourage engagement with the use of CAIM^3–5^. Despite the absence of a universally accepted definition of CAIM, the Cochrane Collaboration has proposed a widely used operational definition, which uses three principal criteria for classification^6,7^. First, it considers whether the intervention originates from a medical system outside the Western allopathic model (that is, considered traditional), thereby including practices such as Chinese medicine. Second, it evaluates whether the intervention is regarded as a standard treatment for a specific condition within the Western allopathic framework. Third, the context in which the intervention is delivered is examined; interventions that are self-administered or provided by practitioners outside conventional medical institutions are more likely to be classified as CAIM. It is important to note that this definition does not incorporate scientific evidence about efficacy as a criterion, thus allowing for the possibility that a CAIM intervention may be supported by robust scientific evidence regarding its effects, although this is not currently the case in the autism literature as our work shows.

The median prevalence of use of any CAIM among autistic people is 54%, with some studies finding a lifetime prevalence as high as 92% (ref. ^2^). This high level of use is explained by a generally positive public perception of the safety and efficacy of CAIM^8,9^. However, many studies and international clinical guidelines indicate a lack of efficacy—and, in some cases, adverse events—for this type of intervention in autism^10,11^. In this perspective, parents of autistic children have identified challenges in navigating the scientific literature on the efficacy/effectiveness and safety of CAIM in autism. In particular, they have highlighted the need for a reliable resource that effectively disseminates this scientific information in an accessible manner, to make informed decisions about the use of CAIM^12^. Therefore, there is an urgent need to synthesize all available evidence on the efficacy and safety of CAIM for autistic individuals and to make this information easily accessible to the wider community, including health professionals, to foster evidence-based shared decision-making. The need for such a tool is reinforced by the positive outcomes of decision-aid tools developed in other fields^13^.

Currently, many meta-analyses on CAIM for autism are available, but it is not uncommon for them to show divergent results regarding the effect of the same intervention for the same clinical population^14^. These discrepancies, which can be caused by different inclusion criteria but also data analysis error or other methodological issues^15^, leave decision-makers, clinicians and individuals with lived experience alike facing conflicting information about the effects of the available interventions. Umbrella reviews are a form of evidence synthesis that addresses this issue by quantitatively synthesizing the information generated by all systematic reviews and meta-analyses on a broad topic, appraising their methodological quality and providing an assessment of the strength and/or quality of the evidence^16,17^. In other words, while the unit of inclusion of systematic reviews and meta-analyses consists of individual primary studies, the unit of inclusion of umbrella reviews consists of systematic reviews and meta-analyses. Umbrella reviews can thus inform clinical decision-makers on the current best secondary, high-level evidence relevant to a specific topic.

In this Article, we conducted an umbrella review investigating the efficacy and safety of CAIMs in autistic individuals. Then, in line with the needs recently expressed by stakeholders^12^, we developed an open-access, interactive online resource (named Evidence-Based Interventions for Autism: Clinical Trials (EBIA-CT), freely accessible at https://ebiact-database.com/) allowing user-friendly access to the results of the umbrella review for each identified participants–intervention–comparator–outcome (PICO). This work adheres to the Umbrella-Review, Evaluation, Analysis, and Communication Hub (U-REACH) framework^18^. The evidence summarized on this website is intended for informational purposes only and should not be used as a replacement for professional medical advice. We strongly recommend that any discussion of these findings be undertaken in consultation with a qualified healthcare provider who is able to assess and appreciate individual clinical circumstances.

## Results

### Results of the searches

**Fig. 1:**
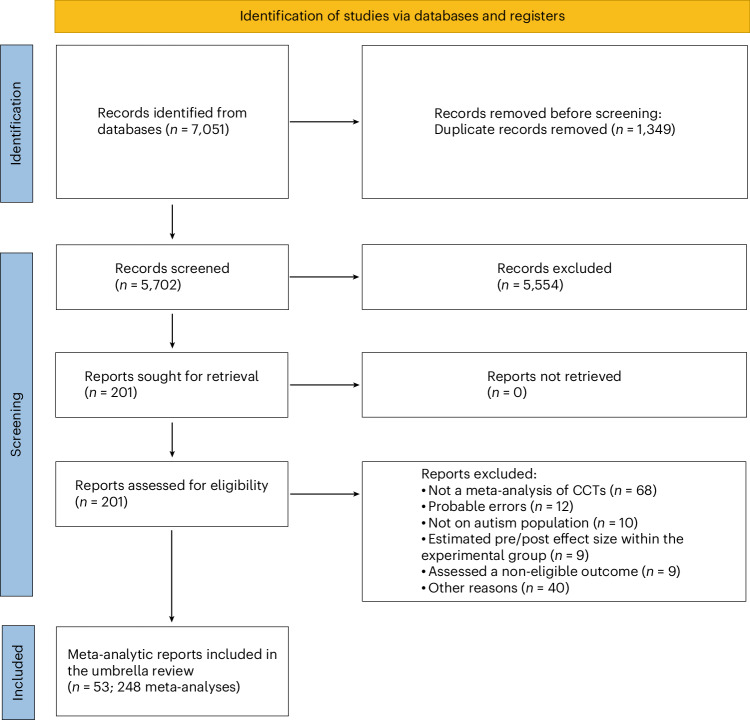
PRISMA flowchart. Selection of the meta-analyses for the umbrella review.

From our initial search, which yielded 7,051 potentially relevant publications (5,702 after removing duplicates), we selected 201 for full-text review and identified 72 meta-analytic reports that met our eligibility criteria (Fig. 1). We then excluded 19 meta-analytic reports: 7 lacked information necessary to replicate calculations, and 12 contained actual or probable data analysis errors. This resulted in a final set of 53 meta-analytic reports included in our analyses. These publications contained a total of 248 different meta-analyses, with each meta-analysis covering a specific combination of PICOs. For example, a single publication could contain several separate meta-analyses examining how the same intervention affects different age groups or different outcomes. Supplementary Information sections 9 and 10 provide full lists of the 53 included and 148 excluded publications.

### Characteristics of the meta-analytic reports retained in the umbrella review

The 53 retained meta-analytic reports examined the effects of at least one of the 19 intervention types (with descriptions of each of them publicly available at https://ebiact-database.com/interventions/) and on at least one of the 19 possible outcomes and were mainly (87%) published after 2018. The 248 meta-analyses synthesized the evidence of more than 200 controlled clinical trials (CCTs) and 10,000 autistic participants and explored 138 unique PICOs. A total of 53 PICOs (38%) were explored in several overlapping meta-analyses. The PICOs with the highest number of overlapping meta-analyses investigated the effects of polyunsaturated fatty acids on disruptive behaviours and social-communication impairment.

### Description of the meta-analyses included in the primary analysis

A total of 138 meta-analyses, derived from 27 reports, were finally included in the primary analysis after discarding overlapping meta-analyses. According to the A MeaSurement Tool to Assess systematic Reviews-2 (AMSTAR-2) scoring, 23 PICOs were evaluated by meta-analyses of high quality (17%), 87 by meta-analyses of low quality (63%) and 28 by meta-analyses of critically low quality (20%). The median number of clinical trials per meta-analysis was 3, and the median number of participants per meta-analysis was 152, with a median percentage of female participants of 14%. A total of 25 meta-analyses (18%) involved very young children, 85 (62%) included school-aged children, 17 (12%) involved adolescents and 11 (8%) focused on adults.

### Results of our primary analysis

**Fig. 2:**
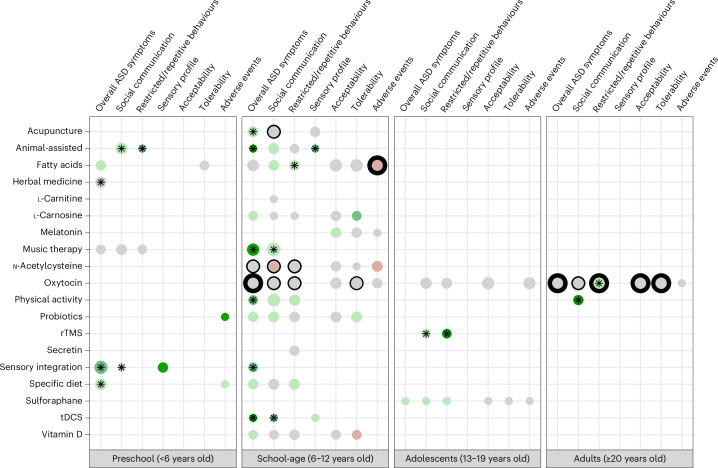
Primary outcome comparisons. Scatter plot indicating, for each PICO combination, the magnitude of the pooled effect sizes (grey: absence of effect (SMD ranging from −0.20 to 0.20, OR/RR ranging from 0.80 to 1.25); green: positive effect; red: negative effect; darker colour indicates larger magnitude), the statistical significance (black stars indicate a two-sided *P* value <0.05), the confidence GRADE rating (no surrounding circle: ‘very low’; light circle: ‘low’; bold circle: ‘moderate’) and the size of the meta-analysis (greater dot width indicates larger meta-analysis). All pooled effect sizes were estimated using random-effects meta-analysis, with no adjustment for multiple testing.

**Fig. 3:**
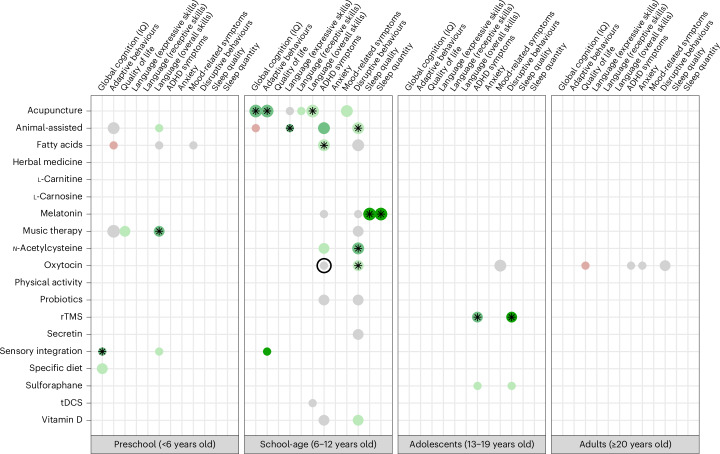
Secondary outcome comparisons. Scatter plot indicating, for each PICO combination, the magnitude of the pooled effect sizes (grey: absence of effect (SMD ranging from −0.20 to 0.20, OR/RR ranging from 0.80 to 1.25); green: positive effect; red: negative effect; darker colour indicates larger magnitude), the statistical significance (black stars indicate a two-sided *P* value <0.05), the confidence GRADE rating (no surrounding circle: ‘very low’; light circle: ‘low’; bold circle: ‘moderate’) and the size of the meta-analysis (greater dot width indicates larger meta-analysis). All pooled effect sizes were estimated using random-effects meta-analysis, with no adjustment for multiple testing.

This section presents meta-analytic results for the primary and secondary outcomes, highlighting findings with a moderate Grading of Recommendations Assessment, Development and Evaluation (GRADE) rating as well as those that exhibit statistically significant and large pooled effect sizes. The GRADE framework assesses the overall quality of evidence supporting meta-analytic estimates based on five criteria: risk of bias of included clinical trials, heterogeneity, publication bias, indirectness and imprecision^19^ (see details in ‘Assessment of the levels of evidence’ section in the Methods). Higher GRADE ratings thus indicate better quality of evidence. Complete results are visually summarized in Figs. 2 and 3 and detailed in Supplementary Information section 11.

#### Main outcomes

We found that oxytocin was the intervention with the highest levels of evidence (that is, the highest GRADE ratings) across all outcomes and age groups (Fig. 2). This intervention demonstrated negligible and not statistically significant efficacy for core autism symptoms across childhood and adulthood (all pooled standardized mean differences (SMDs) ranging from −0.04 to 0.06, and all *P* values exceeding 0.05; all GRADE ratings: from ‘very low’ to ‘moderate’), except for restricted/repetitive behaviours in adults, where a small effect size was found (SMD 0.404, 95% confidence interval (CI) 0.166–0.643; GRADE: ‘moderate’). There were no significant concerns about the acceptability, tolerability or adverse events of oxytocin (all pooled risk ratios (RRs) ranging from 0.72 to 1.30, and all *P* values >0.05; all GRADE ratings: from ‘very low’ to ‘moderate’). The only other effect supported by at least moderate levels of evidence for any of our primary outcomes was the consumption of polyunsaturated fatty acids in school-age children, which was not significantly associated with the occurrence of adverse events (RR 0.676, 95% CI 0.356–1.281; GRADE: ‘moderate’) and did not yield large effects on core autism symptoms (all pooled SMD values <0.50; all GRADE ratings: ‘very low’ or ‘low’).

Notably, a number of interventions demonstrated large effect sizes (SMD ≥0.80) and reached statistical significance, but they were systematically supported by very low levels of evidence. In school-age children, these included music therapy (SMD 0.835, 95% CI 0.316–1.355), animal-assisted interventions (SMD 0.934, 95% CI 0.354–1.515) and transcranial direct current stimulation (tDCS) (SMD 0.844, 95% CI 0.016–1.672) for reducing overall autism symptoms. In adolescents, repetitive transcranial magnetic stimulation (rTMS) appeared to have a large efficacy on restricted/repetitive behaviours (SMD 0.899, 95% CI 0.529–1.269). In adults, physical activity demonstrated potential benefits for social-communication (SMD 0.874, 95% CI 0.486–1.263). Importantly, acceptability, tolerability or adverse events were not evaluated for these interventions.

#### Secondary outcomes

As illustrated in Fig. 3, no intervention presented statistically significant results supported by at least low levels of evidence for the secondary outcomes. Again, several interventions demonstrated large effect sizes with statistical significance, although supported by only very low levels of evidence. In school-age children, melatonin showed promise for improving sleep quality (SMD 1.124, 95% CI 0.349–1.898) and quantity (SMD 1.080, 95% CI 0.413–1.747), while in adolescents rTMS showed promise for reducing disruptive behaviours (SMD 0.940, 95% CI 0.570–1.310). Notably, no statistically significant increase risks in safety outcomes were observed among school-age children using melatonin (with RR values ranging from 0.84 to 1.75 and all *P* values exceeding 0.05; all GRADE ratings: ‘very low’), and safety was not assessed for rTMS use in adolescents.

### Overlapping meta-analyses and EBIA-CT online platform

Among the 53 PICOs that were explored by at least 2 overlapping meta-analyses, the median overlap of the 95% CI of the pooled effect sizes was 50% (Supplementary Information section 12). Only 33 (63%) of PICOs consistently achieved a similar GRADE rating, and only 35 (66%) consistently achieved a similar statistical significance status. Among the PICOs with statistically significant results in our primary analysis and at least one overlapping meta-analysis, we found that ten (62%) presented at least one overlapping meta-analysis with non-statistically significant results. Last, we found that eight PICOs presented a large discrepancy, which we characterized as a substantial difference in either (1) the magnitude of the overlapping estimated effects (that is, the existence of two pooled effect sizes varying by more than SMD ≥0.30 with different statistical significance status, or an average overlap of the 95% CI <20%) or (2) the GRADE status (differing by two points). On the EBIA-CT platform, we enable users to avoid these inconsistencies by systematically presenting the results of the best current scientific evidence, making this platform a convenient hub for the highest-quality scientific evidence.

## Discussion

We conducted a review of the effects of CAIM in autistic individuals, analysing 248 meta-analyses including 200 clinical trials involving over 10,000 participants. Based on this synthesis, we created an interactive user-friendly online platform aimed at disseminating scientific evidence in an accessible format to support healthcare professionals, individuals with lived experience and their families in making informed decisions about CAIM.

Among interventions with an efficacy supported by at least moderate levels of quality of the evidence, only oxytocin showed—for one outcome in one age group—a statistically significant but small effect (SMD <0.50). Several interventions (animal-assisted interventions, melatonin, music therapy, physical activity, rTMS and tDCS) presented statistically significant results with a very large effect size, but the quality of the evidence underpinning the estimation of these effects was very low. The two main factors responsible for these low levels of evidence were imprecision (namely the low number of participants included in a meta-analysis and/or a very wide pooled 95% CI) or limitations (namely the presence of studies at high risk of bias). This finding highlights the need to improve the methodological rigour in the field, to enable a more precise evaluation of the impact of CAIM interventions. In particular, further research on CAIM interventions requires adequately powered studies to precisely estimate their effects while implementing rigorous methodological controls to minimize potential sources of bias. For instance, although blinding may be challenging for outcomes based on daily life reports (for example, quality of life), emerging tools—such as the Brief Observation of Social Communication Change—are being developed to enable blinded assessments of key outcomes in ASD, addressing the limitations inherent in traditional instruments such as the Social Responsiveness Scale and the Vineland Adaptive Behavior Scale. In addition to the generally small effect sizes and low levels of evidence supporting most CAIMs, a critical finding was that safety assessments were missing for most interventions, with less than half of identified CAIMs having any evaluation of acceptability, tolerability or adverse events. This knowledge gap is particularly concerning for interventions with potential adverse effects (such as secretin administration) and represents a major public health challenge given the high frequency of CAIM use in this population^2^. In our view, a key direction for future randomized controlled trials (RCTs) and observational studies will be to explore the safety of CAIMs in autism.

The findings of this study address a critical knowledge gap in the literature regarding CAIM interventions for autism by synthesizing evidence from a substantial body of research previously available in fragmented form only. Previous reviews have typically focused on specific intervention types and did not assess the quality of the generated body of evidence^20–22^. Our approach enables mapping of the evidence across the full spectrum of CAIMs. Notably, the umbrella review methodology used does not allow us to compare efficacy among included interventions (unlike network meta-analyses). However, our umbrella review does establish a unified evidence grading system that allows stakeholders to evaluate the state of evidence for each intervention using consistent, transparent and objective criteria. Moreover, the interactive platform developed addresses the needs from the autism community and the documented disparity between research production and knowledge translation identified by previous studies^12,23,24^. Clinicians and autistic people and their families can collaboratively use this resource to navigate the available evidence for each intervention, including its inherent limitations and the prevailing uncertainty regarding harms, to inform their perspectives about CAIM^25^. This facilitates informed decisions that are tailored to individual needs, preferences and circumstances^26,27^.

From a methodological standpoint, our study revealed a high prevalence of overlapping meta-analyses examining identical populations, interventions, and outcomes. While overlapping analyses can provide valuable independent replication, they often yield inconsistent results. This aligns with previous findings^15,28–30^ and underscores the challenge for healthcare professionals to stay abreast of the best evidence. These inconsistent results are primarily due to differences in the identification of primary studies and the choice of the outcome measure to estimate the effect sizes. Interestingly, our assessment of the quality of the meta-analyses indicated that incomplete searches (which may result in the identification of only a subset of relevant primary studies) and lack of preregistration (which may lead to arbitrary post-hoc decisions about the optimal outcome measure for measuring a given outcome) were among the most common biases observed in the meta-analyses. This suggests that improving key aspects of the meta-analytic methodology (namely adherence to standardized database search guidelines and the development of a protocol before conducting a meta-analysis) may be a promising way to reduce result variability across independent meta-analyses^17^.

The present work should be interpreted in light of its limitations. First, a key methodological decision we made was to assess the levels of evidence using an algorithmic version of the GRADE framework, departing from its original subjective approach. However, as the objective of our work is not to make recommendations, which require subjectivity and are context dependent, we elected to remove the subjectivity from our grading process. This decision aligns with previous large umbrella reviews^31^ and with the typical grading process in network meta-analyses^32^. The algorithmic GRADE assessment used in this Article—based on criteria established in previous studies and developed separately from the present work^32–34^—provides readers with a context-independent algorithmic evaluation of the quality of the evidence, synthesizing a substantial amount of information from each meta-analysis. Second, our analysis was limited to standard pairwise meta-analytic models, which precluded us from making statistical comparisons of the effects of different intervention on the same outcome, or of the effect a single intervention on multiple outcomes. Our choice of extracting clinical trial-level data could have theoretically enabled the use of more advanced meta-analytic models—such as multivariate meta-analysis or network meta-analysis. However, these approaches rely on the critical assumption of transitivity. The substantial methodological heterogeneity across trials in our umbrella review (particularly regarding comparator types, which included placebo, treatment as usual, non-therapeutic interventions and others) meant this assumption could not be reasonably met without proper experimental verification^35^. Such verifications typically extend beyond the scope of umbrella reviews. By maintaining a PICO-level analysis, we adhered to best recommended practices in umbrella reviews prioritizing transparency, interpretability and methodological rigour^36^. Nevertheless, we acknowledge that this approach has inherent limitations, particularly its inability to facilitate comparisons within or between intervention effects. Future research could build on our umbrella review dataset to explore methodological strategies for addressing comparator-related heterogeneity. Such efforts could ultimately inform the feasibility of applying advanced statistical models in this emerging field of evidence synthesis.

In conclusion, this study provides the community with accessible scientific evidence regarding CAIMs for autism, highlighting the current lack of adequate evidence supporting their efficacy and safety. Many studies have examined the effects of various CAIMs for autistic people, but the quality of the evidence generated by these studies is mostly poor. This may lead to imprecise and/or biased estimates of effects. The field requires well-designed, adequately powered RCTs using standardized outcome measures and incorporating comprehensive safety monitoring to facilitate a more nuanced understanding of the benefits and harms associated with each CAIM in the context of autism. Our umbrella review and related platform will be updated on a regular basis to capture the evolution of the evidence in this field. To further enhance the content and features of this platform, future umbrella reviews will be conducted to complete the database (for example, an umbrella review of pharmacological interventions), and collaborative studies will be conducted with healthcare professionals and individuals with lived experience to ensure that the platform meets their needs. Ultimately, the impact of the platform on clinical decision-making will be tested in randomized studies.

## Methods

The umbrella review, based on a publicly available protocol (PROSPERO CRD42022296284), was conducted and reported according to relevant guidelines^16,17,36^, including the U-REACH methodological guidelines^18^ and the Preferred Reporting Items for Overviews of Reviews (PRIOR) reporting guidelines^37^. The PRIOR checklist is available in Supplementary Information section 1.

### Search strategy and eligibility criteria

We searched MEDLINE, Web of Science, Embase, CINAHL and PsycINFO with terms related to two constructs (‘autism’ and ‘meta-analysis’), with no language, publication type or date of publication restrictions, up to 31 December 2023. The list of full search terms is reported in Supplementary Information section 2. Screening of the titles and abstracts, as well as study selection, was performed independently by the first author and several members of the team. Disagreements were resolved by the senior authors (R.D., S.C. and M.S.). References of included studies and Google Scholar were searched to identify additional eligible references, but all relevant reports found with these manual searches were already included in the database searches.

We included systematic reviews coupled with a meta-analysis of both randomized and non-randomized CCTs that assessed the efficacy of any CAIM on both core autism symptoms and key autism-related symptoms in autistic participants of any age. Contrary to what was envisioned in the protocol, we chose to include both randomized and non-randomized controlled trials, rather than including only randomized controlled trials. This choice was made because, in the field of autism, several promising types of intervention—such as long-term or intensive interventions—are difficult to assess in randomized trials. This choice ensures a complete and consistent mapping of the literature regardless of the intervention type. Even if less than 10% of studies were non-randomized studies in our primary analysis, we performed a sensitivity analysis restricted to randomized clinical trials.

A review was considered ‘systematic’ if it was identified as such by its authors and searched at least two scientific databases in combination with explicit inclusion and exclusion criteria. The definition of autism followed that used by primary authors, typically in line with international classifications (Diagnostic and Statistical Manual of Mental Disorders from 3rd to 5th editions, or International Classification of Diseases (ICD) 9 or 10). Based on the mean age of the participants within each meta-analysis, we grouped the presentation of the results into four distinct age groups: (1) preschool children (mean age ranging from 0 to 5 years), (2) school-aged children (6–12 years), (3) adolescents (13–19 years), and (iv) adults (≥20 years). For each meta-analysis, we evaluated whether the average age of the participants from the different CCTs was homogeneous (see our exact criteria in Supplementary Information section 3).

In accordance with the standard classification of the National Institutes of Health National Center for Complementary and Alternative Medicine^6,7^, we identified 19 CAIM types (see the list and complete description at https://ebiact-database.com/interventions/). The prespecified primary outcomes of interest were autism core symptoms (overall symptoms, social communication impairment, restricted/stereotyped/repetitive behaviours and sensory peculiarities) and CAIM-related safety (acceptability (risk of all-cause discontinuation), tolerability (risk of discontinuation due to treatment-related adverse events) and adverse events (risk of experiencing at least one or any specific adverse events)). Secondary outcomes were language skills (overall language, receptive language and expressive language), functioning (overall cognitive functioning, adaptive behaviours and quality of life), disruptive behaviours and psychiatric comorbidities (attention deficit hyperactivity disorder (ADHD), anxiety and emotional/depressive symptoms). We included sleep quality and quantity as post-hoc outcomes, given the significance of sleep for autistic individuals and their families that emerged from the screened papers^38^.

### Data extraction and checking

As described in detail in Supplementary Information section 4, we extracted information regarding the characteristics of clinical trials (for example, risk of bias), participants (for example, mean age) and interventions (for example, dosage) from meta-analytic reports. In instances where the age of the participants was not reported in the meta-analysis, we obtained this directly from the report describing the clinical trial. Similarly, when the estimated effect sizes of individual studies were deemed unplausible (for example, SMD ≥5), we conducted the necessary checks using the metaConvert R package (version 1.0.2)^39^ and excluded meta-analyses containing inaccuracies or errors. More details on data extraction and data quality checks are available in Supplementary Information sections 4 and 5.

### Assessment of the methodological quality

In line with recommendations for umbrella reviews^16–18,36^, we obtained the quality of primary studies by extracting this information directly from the meta-analytic reports. We evaluated the quality of the meta-analyses using the AMSTAR-2 tool^40^. The AMSTAR-2 scoring was performed independently by the first author and several members of the team.

### Overlapping meta-analyses

Following the identification of several overlapping meta-analyses, that is, independent meta-analyses that assessed the same PICO combination, the most recent meta-analysis with the highest methodological quality was selected for the primary analysis, rather than the largest meta-analysis (see more details in ‘Main deviations from the protocol’ section). Then, we assessed the concordance of the results of overlapping meta-analyses, such as the percentage overlap of the 95% CI around the pooled effect size or the agreement on the GRADE ranking, in a secondary analysis. More details on the selection process for overlapping meta-analyses are available in Supplementary Information section 6.

### Assessment of the levels of evidence

When registering the protocol, we did not describe any specific system for assessing the levels of evidence regarding the effects of each intervention. This was because, at that time, there was no consensus on the best approach in umbrella reviews of RCTs, given that the traditional gold-standard framework for meta-analyses of interventions (the GRADE framework) becomes difficult to use when the number of comparisons to grade is high. In this Article, we chose to rely on the algorithmic version of the GRADE framework recently implemented in the metaumbrella R software. These criteria are inspired by standard guidelines for rating the levels of evidence in large evidence syntheses, such as Confidence in Network Meta-Analysis (CINeMA)^32^. For all meta-analyses, the levels of evidence started with a ‘high’ ranking and could then be downgraded depending on the presence of risk of bias, inconsistency, indirectness, imprecision and publication bias (Table 1).

**Table 1:** GRADE criteria (criteria used for the assessment of the levels of evidence, with exact calculations available online)

Risk of bias	• Two downgrades: ≥50% of participants included in high-risk studies. • One downgrade: 25–50% of participants included in high-risk studies.
Heterogeneity	• Two downgrades: substantial discrepancy between the 95% CI and 95% PI (for example, bounds of the 95% CI and 95% PI not of the same sign and in different equivalence ranges). • One downgrade: small/moderate discrepancy between the 95% CI and 95% PI (for example, bounds of the 95% CI and 95% PI of the same sign, but in different equivalence ranges). • When the 95% PI is not estimable, we relied on the value of the I2 statistic and the percentage of studies with results in the opposite direction to the pooled effect size.
Indirectness	• One downgrade: heterogeneous mean age of participants, or more than 25% of participants with unknown types of control group.
Imprecision	• Two downgrades: the 95% CI of the pooled effect size includes both null (SMD 0; RR/OR = 1) and large (SMD ≥0.80; OR/RR ≥ 5) effects, and the meta-analysis does not have the sample size required to detect small effects (eSMD 0.20) with 80% statistical power; or the meta-analysis does not have the sample size required to detect moderate effects (eSMD 0.50) with 80% statistical power • One downgrade: the 95% CI of the pooled effect size included both null and large effects; or the meta-analysis did not have the sample size required to detect small effects (eSMD 0.20) with 80% statistical power.
Publication bias	• One downgrade: P value at the Egger’s or excess of statistical significance test was <0.10; or a proportion of participants included in studies at high risk of reporting bias >50%

PI, prediction interval.

### Development and update of the EBIA-CT platform

To tackle the recognized gap between scientific evidence about CAIM in autism and its accessibility for clinicians and both autistic persons and their families, we developed the EBIA-CT platform (https://ebiact-database.com/) to disseminate our results in a user-friendly manner. Building upon a preliminary version created during our prior work on psychosocial interventions^15^, we significantly enhanced the platform design and features during this project. This improvement process was informed by qualitative feedback from clinicians, stakeholders and researchers, ensuring the platform is user-friendly and effectively disseminates the findings of this umbrella review. Integrating the EBIA-CT platform into our umbrella review methodology allowed us to proactively adhere to the U-REACH guidelines, a framework specifically designed to promote the wide dissemination of umbrella review results. Furthermore, the platform is designed as a ‘living resource’, with planned annual updates for 5 years, following our established methodology and aligning with living review principles. In terms of ethical considerations, no new primary data were collected for this umbrella review or platform development. Therefore, formal ethics committee consultation was not deemed necessary, as is standard practice for projects focused on synthesizing and disseminating existing research findings. The platform is organized into three main sections:

1. Interventions: This section provides comprehensive details about each intervention we identified, including the target population (for example, age range and cognitive functioning level), implementation guidelines and meta-analysis results across age groups. It serves as a valuable resource for autistic individuals and their families seeking information on specific interventions.
2. Preferences: In this section, users can explore age-specific intervention outcomes, providing an overall mapping of the efficacy and safety of all interventions. This section supports informed discussions between clinicians and autistic individuals or their families, facilitating evidence-based decision-making tailored to individual needs.
3. Database: This section provides an overview of the information stored in the platform, and grants access to the raw data from the umbrella review, enabling users to download datasets for external analysis. It is especially useful for researchers identifying knowledge gaps and for guideline developers formulating recommendations.

### Data analysis

As described in more detail in Supplementary Information section 7, all analyses were performed in the R environment (version 4.1.1) using the ‘metaumbrella’ package (version 1.1.0)^41^. We used the SMD as the main effect size measure for the assessment of efficacy, and the RR as the main effect size measure for the assessment of safety (that is, acceptability, tolerability and adverse events). Although we preregistered that the effect measure would be SMD only, we preferred analysing these safety outcomes on their natural effect measures, given their dichotomous nature. For meta-analyses of mean difference and OR, we converted this information for each trial to SMD (respectively RR) before conducting the calculations. Regardless of the metric used (RR or SMD), the direction of the effect was reversed when needed, such that a positive effect size (RR >1 or SMD >0) systematically reflected an improvement, that is, a symptom reduction, a competence improvement or a high safety.

We reran each meta-analysis identified in the review, to ensure consistent calculations and assessments of the level of evidence across meta-analyses. We systematically used a random-effects model with a restricted maximum likelihood estimator, or a Paul–Mandel estimator for *τ*^2^. The CI for the pooled effect size was based on the standard normal quantile method. The *I*^2^, *Q* statistics and *τ*^2^ statistics, as well as the 95% prediction interval, were used to assess heterogeneity. Egger’s regression asymmetry, the excess of statistical significance bias test and the proportion of participants in studies at high risk of reporting bias were used to examine the presence of small study effects^42,43^. When meta-analyses contained dependent effect sizes, we removed this dependence using the standard aggregating approach proposed by Borenstein and colleagues^44^ (Supplementary Information section 8). Because we chose to include both randomized and non-randomized studies, we conducted a sensitivity analysis restricted to randomized controlled trials. However, less than 10% of studies were non-randomized studies in our primary analysis, and the results of this analysis were very similar to those reported in the main manuscript.

### Main deviations from the protocol

We made two important changes to our preregistered protocol. Although we initially planned to select the largest meta-analyses in cases of overlap, we ultimately prioritized the meta-analysis with the highest methodological quality—in line with our willingness to prioritize high-quality evidence for clinical decision-making. In addition, because the methodological landscape of umbrella reviews was still evolving at the time of registration and there were no gold-standard methods for assessing the quality of a very large body of evidence coming from umbrella reviews of RCTs, we did not prespecify an evidence grading system in our protocol. Instead, we relied on recent algorithmic GRADE criteria that have been proposed independently of the current work and implemented in the main umbrella review software^34^. Although these departures were made to increase methodological rigour, they represent post-hoc decisions that should be taken into account when interpreting our results and reflect the recent nature of the field of umbrella reviews.

### Reporting summary

Further information on research design is available in the Nature Portfolio Reporting Summary linked to this article.

## Supplementary information

Supplementary Information Supplementary Information sections 1–12.

Reporting Summary

## Data Availability

The datasets generated and/or analysed during the current study are publicly available via GitHub at https://github.com/CorentinJGosling/EBIACT_CAM_2024.

## Code availability

Additional information on the results and R code supporting data analysis is publicly available at https://corentinjgosling.github.io/EBIACT_CAM_2024.
