# Supplementary material for: Complementary, alternative and integrative medicine for autism: an umbrella review and online platform
Source: Nat Hum Behav. 2025 Aug 28;9(12):2610–9. doi: 10.1038/s41562-025-02256-9 (PMC12727493; doi:10.1038/s41562-025-02256-9)
Supplement: Supplementary file 1 — Supplementary Information sections 1–12. [file 41562_2025_2256_MOESM1_ESM.pdf]

nature human behaviour

Supplementary information https://doi.org/10.1038/s41562-025-02256-9

Complementary, alternative and integrative medicine for autism: an umbrella review and online platform

In the format provided by the authors and unedited

S1. PRIOR checklist 2
https://corentinjgosling.github.io/EBIACT_CAM_2024/#S1_PRIOR_checklist 2
S2. Search strategies 2
https://corentinjgosling.github.io/EBIACT_CAM_2024/#S2_Search_strategies 2
S3. Classification of age groups 2
https://corentinjgosling.github.io/EBIACT_CAM_2024/#S3_Classification_of_age_groups 2
S4. Data extraction 2
https://corentinjgosling.github.io/EBIACT_CAM_2024/#S4_Data_extraction 2
S5. Data extraction quality checks 2
https://corentinjgosling.github.io/EBIACT_CAM_2024/#S5_Data_quality_checks 2
S6. Details on overlapping meta-analyses 2
https://corentinjgosling.github.io/EBIACT_CAM_2024/#S6_Strategy_overlapping 2
S7. Data analytic strategy 2
https://corentinjgosling.github.io/EBIACT_CAM_2024/#S7_Data_analytic_strategy 2
S8. Dependent effect sizes 2
https://corentinjgosling.github.io/EBIACT_CAM_2024/#S8_Dependent_effect_sizes 2
S9. Included studies 2
https://corentinjgosling.github.io/EBIACT_CAM_2024/#S9_Included_studies 2
S10. Excluded studies 3
https://corentinjgosling.github.io/EBIACT_CAM_2024/#S10_Excluded_studies 3
S11. Primary analysis 3
https://corentinjgosling.github.io/EBIACT_CAM_2024/#S11_Main_analysis 3
S12. Overlapping meta-anlyses 3
https://corentinjgosling.github.io/EBIACT_CAM_2024/#S12_Overlapping 3

S1. PRIOR checklist

https://corentinjgosling.github.io/EBIACT_CAM_2024/#S1_PRIOR_checklist

S2. Search strategies

https://corentinjgosling.github.io/EBIACT_CAM_2024/#S2_Search_strategies

S3. Classification of age groups

https://corentinjgosling.github.io/EBIACT_CAM_2024/#S3_Classification_of_age_groups

S4. Data extraction

https://corentinjgosling.github.io/EBIACT_CAM_2024/#S4_Data_extraction

S5. Data extraction quality checks

https://corentinjgosling.github.io/EBIACT_CAM_2024/#S5_Data_quality_checks

S6. Details on overlapping meta-analyses

https://corentinjgosling.github.io/EBIACT_CAM_2024/#S6_Strategy_overlapping

S7. Data analytic strategy

https://corentinjgosling.github.io/EBIACT_CAM_2024/#S7_Data_analytic_strategy

S8. Dependent effect sizes

https://corentinjgosling.github.io/EBIACT_CAM_2024/#S8_Dependent_effect_sizes

S9. Included studies

https://corentinjgosling.github.io/EBIACT_CAM_2024/#S9_Included_studies

S10. Excluded studies

https://corentinjgosling.github.io/EBIACT_CAM_2024/#S10_Excluded_studies

S11. Primary analysis

https://corentinjgosling.github.io/EBIACT_CAM_2024/#S11_Main_analysis

S12. Overlapping meta-anlyses

https://corentinjgosling.github.io/EBIACT_CAM_2024/#S12_Overlapping
